# Emotional Dysregulation and Adaptive Functioning in Preschoolers With Autism Spectrum Disorder or Other Neurodevelopmental Disorders

**DOI:** 10.3389/fpsyt.2022.846146

**Published:** 2022-04-11

**Authors:** Chiara Davico, Daniele Marcotulli, Valentina Francesca Cudia, Luca Arletti, Ada Ghiggia, Barbara Svevi, Chiara Faraoni, Federico Amianto, Federica Ricci, Benedetto Vitiello

**Affiliations:** ^1^Section of Child and Adolescent Neuropsychiatry, Department of Public Health and Pediatric Sciences, University of Turin, Turin, Italy; ^2^Department of Psychology, University of Turin, Turin, Italy; ^3^Department of Neuroscience, University of Turin, Turin, Italy

**Keywords:** autism (ASD), neurodevelopmental disorder, adaptive functioning, emotional dysregulation, developmental delay

## Abstract

**Aim:**

Emotional dysregulation (ED), defined by deficits in the ability to monitor and modulate the valence, intensity, and expression of emotions, is typically expressed with irritability, tantrums, mood fluctuations, and self-harm in young children with autism spectrum disorder (ASD). Although ED does not represent a diagnostic feature of ASD, its manifestations are an important contributor to functional impairment and clinical referral. This study aims to examine the relationship between ED and adaptive functioning in preschoolers clinically referred for ASD or other neurodevelopmental disorders.

**Methods:**

A sample of 100 children (74% males, mean age 39.4 ± 12.3 months), consecutively referred to a university clinic for neurodevelopmental disorders, received clinical assessments of psychopathology with the CBCL and the Autism Diagnostic Interview-Revised, of ED- with the CBCL-Attention, Anxious/Depressed, and Aggression index (CBCL-AAA), of autism symptom severity with the ADOS-2 Calibrated Severity Score (ADOS-CSS), and of global developmental/cognitive delay (GDD) with the WPPSI-IV or other age-appropriate standardized scales. Adaptive functioning was measured with the ABAS-II. Sixty-five children met DSM-5 criteria for ASD. Multivariate regression models were applied to evaluate the relative contribution of ED, ASD severity and GDD to the ABAS-II general (GAC), conceptual (CAD), social (SAD), and practical (PAD) adaptive functioning domains.

**Results:**

Overall (*n* = 100), lower adaptive functioning was associated with higher CBCL-AAA (*p* = 0.003), higher ADOS-CSS (p < 0.001), and presence of GDD (*p* = 0.023). In the ASD group (*n* = 65), worse CAD was predicted by GDD (*p* = 0.016), and worse SAD and PAD by higher ADOS-CSS (*p* = 0.032) and ED (*p* = 0.002). No sex differences were detected in the study variables.

**Conclusion:**

Together with the severity of global developmental delay and of autism symptoms, ED is a significant contributor to impairment in adaptive functioning among young children with a neurodevelopmental disorder and, in particular, with ASD. ED could represent a specific target for early interventions aimed at enhancing adaptive functioning in early childhood.

## Introduction

Emotional dysregulation (ED), defined as a deficit in the ability to monitor and modulate the valence, intensity, and expression of emotions, is typically expressed with mood instability, irritability, tantrums, and self-harm in young children with autism spectrum disorder (ASD) (1–4). Although ED is not among the diagnostic criteria of ASD, its manifestations seem to be an important contributor to functional impairment and clinical referral (5); furthermore it appears to be related to ASD core symptoms (5). Indeed, children with ASD often have problems in recognizing and managing emotions, inhibiting emotional reactions, delaying gratification, and tolerating transitions (6).

Emotional dysregulation represents a transdiagnostic risk factor for mental health disturbances both in the general population (7, 8) and in ASD (9, 10), and could have relevance as a possible target for developing specific therapeutic interventions (11). ED occurs when the ability to control the intensity, duration, and type of the emotions experienced is impaired, resulting in negative affectivity or irritability. Emotion regulation typically occurs in an adaptive way with the capacity to upregulate positive emotions and down regulate negative ones. There are many strategies that can be used to down regulate negative emotions: some of them, such as avoidance, denial, and negative rumination, are maladaptive and associated with poorer outcome in term of anxiety and depression, while others, such as cognitive reappraisal, problem solving, and acceptance, are more adaptive (12). In particular, the flexibility in being able to adopt more than one strategy seems to be especially adaptive (12).

When emotional regulation is not effective, intense emotions interfere with social interaction. Children with such difficulties tend to engage in peer conflicts and, consequently, to be less accepted by others and to experience negative feed-back in relationships, with consequent feelings of exclusions (13). Indeed, in longitudinal studies of children with ASD, emotion regulation abilities seem to be associated with subsequent prosocial behaviors (14). Ineffective emotional regulation impairs also the ability to perform goal oriented activities and are therefore involved in learning processes (15).

The ability to regulate emotions grows with age, being less effective in preschoolers than in older children, is influenced by puberty with decreased regulation during adolescence (16), and becomes more effective in adulthood (17). Parenting style is a key factor for developing good self emotion strategies during childhood (18), as is also the social context in which the child grows (19). Sex differences in emotion regulation strategies have been reported across different phases of development in the general pupulation (19). For example, a study reported that females engaged in more social support seeking strategies and dysfunctional rumination, whilst males employed more passivity, avoidance, and suppression strategies (20).

People with ASD seem to be at higher risk of having impaired emotional regulation because of poor emotional awareness and low competence in emotional language, poor flexibility, high sensitivity to change and to environmental stimulation (21). Due to these deficits, emotions become difficult to manage, negative emotions tend to last longer and can lead to prolonged ruminations after social setbacks and to intense reactions to social rejection, with consequent need of intervention from others to externally regulate emotions. In younger kids, ED can be expressed with externalizing behavior, such as aggression, irritability, tantrums, self harm, and “meltdowns” or “shutdowns.” If left untreated, ED in children with ASD tends to persist over time (22).

As ED underlies several behavioral problems in ASD, therapeutic interventions aimed at empowering the ability to recognize and regulate emotions might be an effective way of addressing behavioral problems (21). The social adaptive domain (SAD) has been found to be more impaired than other domains in ASD even after accounting for sex, age, and cognitive development (23, 24). Although there is wide heterogeneity in the level of functioning among people with ASD, cross sectional studies have documented an inverse correlation between autism symptom severity and adaptive functioning (25). Moreover in ASD people there is often a pronounced discrepancy between cognitive abilities and adaptive functioning profile, and this seems to be more pronounced in individuals with older age, higher IQ and higher social-communication symptoms (26). Compared with individuals with the same age and IQ, people with ASD have a lower overall adaptive functioning profile (27). Results indicated that impairment in adaptive behavior within the domain of socialization skills remains a distinctive factor of ASD (24). Adaptive functioning in children with ASD is positively associated with a family’s quality of life (28).

More in detail, the ABAS-II Social Adaptive Domain score is derived from the scores of the two subscales: play and social abilities. Since ABAS-II examines “real-world” functions, it provides an index of the child’s competency in everyday contexts, such as the ability to engage in leisure activities by its own, to interact with others while playing, to ask for adult’s involvement while playing, to share emotions, and to exhibit social engagement, stages of according to different developmental age.

To our knowledge no study has analyzed the relative contribution of ED to adaptive functioning in ASD in very young children.

This study aimed to examine the relationship between ED and adaptive functioning in preschoolers clinically referred for ASD or other neurodevelopmental disorders, while taking into account autism symptoms, sex, and presence of global developmental/cognitive delay.

## Materials and Methods

### Design and Participants

This was a cross-sectional study of 100 consecutively referred children undergoing a diagnostic assessment for developmental disorder. All children under 6 years of age referred to the University of Turin-Pediatric Hospital Regina Margherita Outpatient Service for Neurodevelopmental Disorders between 1 July 2018 and 31 October 2021 received a comprehensive diagnostic evaluation. The children were referred by primary care pediatricians or directly brought in by their parents for suspicion of a neurodevelopmental disorder because of difficulties in communication and social interaction or behavioral problems. Neuropsychological and psychopathological evaluations were conducted by trained developmental psychiatrists and neuropsychologists. Main caregiver psychometric questionnaire was used for research purposes. Parents gave informed permission to participate, and all the procedures were approved by the local ethics committee. All the children received the following assessments.

### Assessments

#### General Psychopathology and Autism Spectrum Disorder

The Child Behavior Checklist 1.5-5 (CBCL) (29), Italian version (30), a 100-item parent-report checklist assessing emotional and behavioral problems in 1.5 to 5-year-old children, was completed by the main caregiver. On the CBCL problem behaviors are rated on a three-point scale (0 not true, 1 somewhat/sometimes true, or 2 very/often true). Raw scores are converted into T-scores, obtaining eight syndromic scales (emotionally/reactive, anxious/depressed, somatic complaints, withdrawal, sleep problems, attention problems, aggressive behavior, and other problems), which contribute to two general dimensions (internalization and externalization) and one total score. T-scores are computed for each scale score and for the total score. T-scores between 65 and 70 are considered borderline, and those above 70 clinically abnormal.

The Autism Diagnostic Interview-Revised (ADI-R) (31) was administered. Autistic symptoms were assessed with the Autism Diagnostic Observation Schedule-2 (ADOS-2) (32), which is a semi-structured direct assessment of communication, social interaction, and play or imaginative use of materials for individuals with a suspected diagnosis of ASD. The ADOS consists of four or five modules designed for children and adults with different language levels, ranging from non-verbal to verbally fluent. The ADOS was administered and scored by trained clinicians who had demonstrated clinical proficiency in the use of this instrument.

The diagnosis of ASD was made according to the DSM-5 criteria (2), using the gold standard assessment procedure comprehensive of ADOS-2 and ADI-R (33). In this study, children younger than 3 years of age who met criteria for being “at risk” for ASD were included in the ASD group.

The severity of the autism symptoms was measured with the calibrated severity score (CSS) (34, 35) of the ADOS-2. The total CSS was calculated for the Toddler Module of ADOS-2 based on Esler et al. (36) to facilitate a direct comparison with other modules of the ADOS-2.

#### Emotional Dysregulation

Emotional dysregulation was measured using the Attention, Aggression, and Anxious/Depressed scales of the CBCL (CBCL-AAA), which consider the affective, behavioral, and cognitive dimensions of ED. The CBCL-AAA construct was first used to define the Deficient Emotional Self-Regulation (DESR) with scores between 1 and 2 standard deviations (SD) above the mean (37), while an elevation above 2 SD characterizes the emotional dysregulation profile (38). First used with the 6–18 CBCL, the CBCL-AAA profile has been applied also to preschool samples (39, 40). The CBCL-AAA score is computed by the sum of the Attention, Aggression, and Anxious/Depressed CBCL T scores.

#### Cognitive or Global Development

According to age, expressive language level, and the child’s ability to engage and cooperate with the examiner, standardized tests were used to assess cognitive and global development level. Instruments included the Griffiths III or Bayley-II developmental scales, Leiter International Performance Scale-II edition or the WPPSI-IV (Wechsler Preschool and Primary Scale of Intelligence-III Edition) (41–44). All of these measures use the same standard scores (SS = 100) and standard deviations (SD = 15). Cognitive or developmental scores were categorized according to the corresponding percentile. Intellectual disability and or global developmental delay (GDD) was defined by an intelligent quotient (IQ < 70) or a general quotient (GQ) lower than the 3rd centile.

#### Adaptive Functioning

Adaptive functioning, defined as the set of conceptual, social and practical skills learned and used in everyday life, was measured with the Adaptive Behavior Assessment System-Second Edition (ABAS-II) (45). On the Parent Form (Ages 0–5), Italian version (46), the parent rates how frequently the child performs a correct behavior when necessary and without help. The items, rated on a Likert scale ranging from 0 to 3 (from 0 = “not able to” to 3 = “able to do it and always performs it when needed”), are grouped into 10 skill areas: communication, use of environment, preschool competences, domestic behavior, health and safety, play, self-care, self-control, social abilities, and motility. Skill area scores are norm-referenced scaled scores with a mean of 10 and a standard deviation of 3, and are combined into three composite scores: Conceptual Adaptive Domain (CAD), Social Adaptive Domain (SAD), and Practical Adaptive Domain (PAD), in addition to an overall General Adaptive Composite (GAC). Composite scores are norm-referenced standard scores with a mean of 100 and SD of 15.

### Statistical Analyses

Statistical analyses were performed using the statistical programming language R (version 4.0.5) (47). Descriptive statistics was applied to the sociodemographic and clinical data. Continuous variables were described by mean and SD, and categorical data as percentages. z-test was used to evaluate differences between proportions of categorical variables. Group differences for continuous variables were assessed with a two-tailed Mann Whitney U-test. A *p*-value < 0.05 was considered statistically significant.

Because of multiple testing with correlated subscales, multivariate regressions were used to determine the association of ED with adaptive functioning. The multivariate models considered the three ABAS-II subscales as dependent variables. All models were also adjusted by ADOS-CSS and a dichotomous variable for global developmental delay/intellectual disability. Pillai’s trace statistic was used to evaluate significance of the multivariate models. The associated univariate multivariable effects were then analyzed and *p*-values corrected using the Benjamini-Hochberg procedure.

Since no significant difference between males and females in ED, CSS, and adaptive functioning were detected in the whole sample, we did not include sex in the models. Also, as the sample was homogeneously composed by preschoolers, we did not adjust the models by age.

The same multivariate and univariate multivariable models were used for evaluating the impact of ED, ADOS-CSS, and GDD on adaptive functioning in the ASD group.

## Results

The sample consisted of 100 patients, of whom 74 were males (74%). Mean age at first evaluation was 39.43 months (SD 12.25, range 18–70 months) (Table 1). Sixty-five children (65%) met the specified criteria for inclusion in the ASD group. Sixty children (60%) scored under the 3rd percentile at cognitive or developmental tests and were therefore categorized as having intellectual disability/developmental delay (GDD).

**TABLE 1:** Demographics and clinical characteristics.

	All sample (*n* = 100)	Patients with ASD (*n* = 65)	Patients without ASD (*n* = 35)	*p* value
Male, *n* (%)	74 (74.0)	51 (78.5)	23 (65.7)	0.25
Mean age at evaluation, months, mean (SD)	39.43 (12.25)	37.21 (12.23)	43.54 (11.33)	**0.01**
Global developmental delay, *n* (%)	60 (60.0)	33 (50.8)	28 (80.0)	**<0.01**
**ABAS-II, mean (SD)**				
General adaptive composite (GAD)	71.45 (18.66)	65.96 (16.76)	81.62 (17.91)	**<0.01**
Conceptual adaptive (CAD)	69.40 (18.38)	68.00 (16.36)	72.00 (21.66)	0.50
Social adaptive (SAD)	78.56 (18.95)	74.26 (16.04)	86.54 (21.46)	**<0.01**
Practical adaptive (PAD)	76.82 (19.08)	71.30 (16.05)	87.05 (20.25)	**<0.01**
ADOS-CSS, mean (SD)	4.95 (3.16)	6.75 (2.37)	1.60 (0.88)	**<0.01**
**CBCL Problem Scales, mean (SD)**				
Emotionally/reactive	57.8 (9.60)	58.52 (10.12)	56.45 (8.51)	0.25
Anxious/depressed	57.04 (9.33)	56.97 (9.53)	57.15 (9.06)	0.73
Somatic complaints	56.92 (8.98)	57.90 (9.72)	55.1 (7.17)	0.09
Withdrawal	64.16 (12.13)	66.83 (12.86)	59.2 (8.84)	**<0.01**
Sleep problems	57.2 (8.88)	56.93 (7.97)	57.7 (10.46)	0.95
Attention problems	60.18 (8.31)	61.71 (8.22)	57.32 (7.81)	**<0.01**
Aggressive behavior	55.89 (7.50)	56.60 (6.91)	54.57 (8.44)	0.10
Internalizing	56.51 (11.01)	57.61 (10.66)	54.47 (11.52)	0.13
Externalizing	54.20 (9.74)	55.02 (9.98)	52.68 (9.24)	0.12
Total	54.92 (11.20)	55.40 (11.38)	54.02 (10.95)	0.46
AAA	173.11 (19.85)	175.30 (19.32)	169.05 (20.47)	0.05

*ABAS-II, adaptive behavior assessment system-second edition; CBCL, child behavior check-list; ADOS, autism diagnostic observation schedule; CSS, calibrated severity score; GAC, general adaptive composite; CAD, conceptual adaptive domain; PAD, practical adaptive domain; SAD, social adaptive domain; AAA, attention, aggression, anxious/depressed score; SD, standard deviation. Statistically significant values are bold.*

On the CBCL, mean scores were: 56.51 ± 11.01 on the internalizing scale and 54.20 ± 9.74 on the internalizing scale, with a total score of 54.92 ± 11.20. Detailed subscales of CBCL are reported in Table 1, with comparison between the ASD and non-ASD groups.

The mean CBCL-AAA T-score of the total sample was 173.11 ± 19.85), with no statistically significant difference between the ASD (175.30 ± 19.32) and the non-ASD group (169.05 ± 20.47) (*p* = 0.054). No sex difference was found in our sample with regard to adaptive functioning, ADOS CSS, CBCL AAA, and GDD (Table 2).

**TABLE 2:** Sample characteristics by sex (*n* = 100).

	Males (*n* = 74)	Females (*n* = 26)	*p* value
Mean age at evaluation, months, mean (SD)	39.69 (11.81)	38.69 (13.64)	0.59
ASD, *n* (%)	51 (68.9)	14 (53.85)	0.25
Global developmental delay *n*, %	28 (37.84)	12 (46.15)	0.61
**ABAS, mean (SD)**			
GAC	70.12 (19.07)	75.23 (17.26)	0.24
CAD	68.40 (19.47)	72.23 (14.24)	0.39
SAD	77.53 (19.82)	81.50 (16.23)	0.50
PAD	76.19 (19.80)	78.61 (17.14)	0.45
ADOS-CSS, mean (SD)	5.15 (3.12)	4.38 (3.27)	0.25
CBCL-AAA, mean (SD)	173.05 (19.72)	173.31 (20.64)	1.00

*ABAS-II, adaptive behavior assessment system-second edition; CBCL, child behavior check-list; ADOS, autism diagnostic observation schedule; CSS, calibrated severity score; GAC, general adaptive composite; CAD, conceptual adaptive domain; PAD, practical adaptive domain; SAD; social adaptive domain; SD, standard deviation.*

The ABAS mean General Adaptive Composite score (GAC) of the sample was 71.45 ± 18.66, conceptual adaptive domain (CAD) mean score was 69.40 ± 18.38, social adaptive domain (SAD) mean score was 78.56 ± 18.95, and practical adaptive domain mean score (PAD) was 76.82 ± 19.08.

The mean GAC score of patients who received a diagnosis of ASD (65.96 ± 16.76) was lower that in patients without ASD (81.62 ± 17.91; *p* < 0.0001). Scores for DAS (74.26 ± 16.04 in ASD vs. 86.54 ± 21.46 in non-ASD, *p* = 0.0004) and DAP (71.30 ± 16.05 in ASD vs. 87.05 ± 20.25 in non-ASD, *p* < 0.0001) were also lower in children with ASD. No significant difference was found between the ASD and non-ASD with regard to CAD (*p* = 0.05, Table 1).

The mean ADOS CSS score of the whole sample was 4.95 ± 3.16). As expected, the mean ADOS CSS in ASD (6.75 ± 2.37) was higher than in non-ASD (1.60 ± 0.88; *p* < 0.0001).

The association between ED and overall adaptive functioning was then evaluated. The model was adjusted by ADOS CSS and GDD. Higher ED was associated with lower overall adaptive functioning (*p* = 0.003). Significant associations between ADOS CSS and adaptive functioning (*p* < 0.001) and GDD and adaptive functioning (*p* = 0.023) were also revealed (Table 3).

**TABLE 3:** Multivariate and univariate multivariable analysis for the entire (*n* = 100) and the ASD (*n* = 65) sample, with ADOS-CSS, CBCL-AAA, and GDD as independent variables.

Multivariate multivariable analysis for the entire sample (*n* = 100), Type II MANOVA General adaptive composite (GAC)						
		Pillai test				*p* value
ADOS CSS		0.137				**0.003**
CBCL AAA		0.158				**<0.001**
GDD		0.096				**0.023**

**Univariate multivariable analysis for the entire sample (*n* = 100)**						

	**CAD**		**SAD**		**PAD**	
	
	**Est.**	**Adjusted *p***	**Est.**	**Adjusted *p***	**Est.**	**Adjusted *p***

ADOS- CSS	−0.37	0.542	−1.34	**0.026**	−1.85	**0.001**
CBCL-AAA	−0.18	**0.046**	−0.28	**0.002**	−0.32	**<0.001**
GDD	−11.34	**0.008**	−9.93	**0.011**	9.43	**0.011**

**Multivariate multivariable analysis for ASD (*n* = 65), Type II MANOVA**						

		**Pillai test**				***p* value**

ADOS- CSS		0.14				**0.032**
CBCL-AAA		0.15				**0.023**
GDD		0.11				0.066

**Univariate multivariable analysis for ASD patients only (*n* = 65)**						

	**CAD**		**SAD**		**PAD**	
	
	**Est.**	**Adjusted *p***	**Est.**	**Adjusted p**	**Est.**	**Adjusted *p***

ADOS-CSS	−1.68	0.055	−1.88	**0.032**	−2.30	**0.011**
CBCL-AAA	−0.15	0.121	−0.26	**0.011**	−0.24	**0.015**
GDD	10.52	**0.016**	6.51	0.099	7.72	0.050

*GDD, global developmental delay; ABAS-II, adaptive behavior assessment system-second edition; CBCL, child behavior check-list; ADOS, autism diagnostic observation schedule; CSS, calibrated severity score; GAC, general adaptive composite; CAD, conceptual adaptive domain; PAD, practical adaptive domain; SAD; social adaptive domain. Statistically significant values are bold.*

To study the impact of ED on the diverse domains of adaptive functioning, we analyzed a univariate multivariable model for each adaptive functioning domain (Table 3). Lower CAD levels were predicted by the presence of GDD (estimate mean difference −11.34, *p* = 0.008) and by higher ED (estimate −0.18, adjusted *p* value = 0.046), while both higher ED (estimate −0.28, adjusted *p* value = 0.002) and ADOS CSS (estimate −1.34, adjusted *p* value 0.026) were associated with lower SAD. Higher levels of ADOS CSS (estimate −1.85, adjusted *p* value = 0.001) and ED (estimate −0.32, adjusted *p* value < 0.001), and the presence of GDD (estimated mean difference 9.43, adjusted *p* value = 0.011) were all significant predictors of worse PAD.

Likewise, when considering only children with ASD, higher ED was associated with lower adaptive functioning levels. Also the ADOS CSS was associated with overall adaptive functioning. With regard to the three adaptive functioning domains (Table 3), CAD was only predicted by GDD (estimated mean difference 10.52, adjusted *p* value = 0.016). ADOS CSS (estimate −1.88, *p* value 0.032) and ED (estimate −0.28, adjusted *p* value 0.002) were both found to be associated with worse SAD. ADOS CSS (estimate −2.30, adjusted *p* value 0.011) and CBCL AAA (estimate −0.24, adjusted *p* value = 0.015) predicted lower PAD.

## Discussion

Prior research has suggested that ED in children with ASD is a significant problem that is likely to underlie behavioral difficulties, such as tantrums, self-harm and irritability, and thus to contribute to functional impairment. The pathogenetic relevance and the mechanisms of the relationship between ED and clinical symptoms are still a matter of study, and investigation into ED in ASD children is ongoing. This study aimed to contribute to the research in this area by examining the relationship between ED and functional impairment, and by measuring different domains of adaptive functioning in preschoolers clinically referred for suspected ASD. To our knowledge, this study has been the first to evaluate autism symptoms, emotional dysregulation and adaptive functioning in preschoolers with ASD and other neurodevelopmental disorders. The clinical characteristics of the study sample are consistent with those expected in this population, with a predominance of the male sex and about two-third of the referred children meeting criteria for ASD or being at high risk for ASD (48).

Most studies have reported prevalence rates of ED between 50 and 60% in ASD (49–52), which are higher than those reported in typically developing children (22, 53–55) or in children with other neurodevelopmental disorders (54). However, a recent large study in clinically referred children aged 6–18 years reported higher rates of ED among non-ASD compared to ASD children, using the CBCL-AAA as a measure of ED (56). In our sample, no significant differences were found between ASD and non-ASD regarding ED, even if ED was numerically higher in ASD, with a *p* = 0.05 (Table 1) that closely missed the pre-specified alpha threshold (p < 0.05). Clinically referred children, like the preschoolers included in our sample, have been generally found to have high levels of ED. Among samples of clinically referred children for a suspicion of ASD, about 60% (57) receive an ASD diagnosis, while the others are very often diagnosed with language disorders, global developmental delays or having inattention, hyperactivity, and disruptive behaviors. The relationship between ADHD (58–61), developmental delays (62) or other disorders (63) and emotional has been extensively reported. Moreover boys with specific language impairment, have been reported to have lower emotional regulation abilities than typically developing children (64). However, the current literature on ED in clinically referred preschoolers remains rather limited.

The close relationship between ED and core autism symptoms has led some to suggest that ED should be considered among the ASD features (50). But, ED is also a well known transdiagnostic risk factor for many mental disorders in children and adolescents, such as, among the others, ADHD, mood disorders, posttraumatic stress disorder, non-suicidal self-injury, and disruptive mood dysregulation disorder (65). In fact, the CBCL-AAA index was initially studied as a measure of high risk for bipolar disorder in adolescence (66–69), and subsequently as a risk factor for other psychopathology associated with suicidality in adults (68, 70). CBCL-AAA has been applied also to the preschooler population, using the 1 and 1/2 –5 years old version of the CBCL (39, 40). In non-clinical preschoolers population there was an association between higher scores on CBCL AAA and psychiatric symptomatology, lower adaptive functioning, temperament and maladaptive parenting (39).

A specific impairment in the social adaptive functioning domain (SAD) had been reported to be distinctive of ASD compared with other neurodevelopmental disorders (24). In our sample, however, we found that ASD was accompanied by a general impairment in multiple domains of functioning, and not just SAD (Table 1). This is consistent with what was recently reported in an Italian preschooler sample (71). Previous reports have confirmed that toddlers (younger than 2 years old) with ASD have a lower adaptive functioning compared to children with developmental delay (72).

In our study, considering both the overall sample and also ASD sample, higher ED was associated with lower adaptive functioning levels (Table 3). Social and practical domains are significantly influenced by both autism symptoms severity, as expected (25), and by ED, as hypothesized for this study. The relationship between emotional regulation strategies and subsequent social competences is well documented. Berkovits et al. analyzed a group of 4–7 years old children with ASD and found that ED was strongly related to social and behavioral functioning but was largely independent of IQ (5). Reyes et al. compared two samples of ASD children and typical developing peers (3–7 years), and found that in ASD better social skills were associated with greater emotional regulation and increased expression of emotions. Better emotional regulation was linked to fewer peer problems and more prosocial behaviors (22).

We didn’t find any sex difference in ED or in other clinical variables. This could be due to the rather small representation of females in the study sample and/or to referral bias whereby females with high ED might have been more likely to be clinically referred. Few data on sex differences in emotional regulation among ASD people are available, but scarcely comparable to our population (55, 73). Further studies on sex differences in preschoolers with ASD are needed to evaluate different trajectories of development, and so identify vulnerable children.

The results of this study are clinically important because they document the relevance of ED to adaptive functioning in young children and thus provide support to efforts to develop emotional regulation strategies as a possible means of ultimately improving functioning. In typical development, good parental scaffolding helps children overcome frustration and improve emotion regulation. ASD children too can benefit from parental scaffolding but they seem to have difficulty in internalizing and generalizing co-regulatory support. This is consistent with the clinical experience that children with ASD benefit from the presence of one-to-one support for regulatory management in education settings well into middle childhood and beyond (74). Parent-mediated early interventions targeted to ED in preschool years could therefore be considered as a means of improving functioning. More research is needed to better understand the relationship between ASD symptoms, ED, and parenting style within the social context.

## Limitations

This study has several limitations. First, the cross sectional design precludes the possibility of assessing prospectively the interplay between ED and adaptive functioning or to evaluate the developmental trajectories of these constructs over time. Second, the study sample included only clinically referred children without a comparison group of typically developing children. Third, the male preponderance, while being consistent with the epidemiology of the disorder, limits inferences to female patients. Fourth, both the ED and adaptive functioning ratings were provided by the same informant (main caregiver), thus limiting somewhat the external validity of the associations. Finally, no systematic information on the social context was available. Further studies with larger samples and prospective design are needed to confirm and better clarify our findings.

## Conclusion

Higher levels of ED were associated with lower adaptive functioning in all the domains evaluated, regardless of the severity of the autistic symptoms or the presence of GDD. Decreasing ED in preschoolers with ASD or other neurodevelopmental disorders may be an effective way of improving their overall adaptive functioning, including prosocial behavior, ability to focus on goal oriented activities, and academic achievement. In particular, since a child’s ability to regulate emotions is modeled by parental scaffolding, parent-mediated early interventions may be an effective approach to improving the developmental trajectory of children with high levels of ED.

## Data Availability Statement

The raw data supporting the conclusions of this article can be made available by the authors upon qualified request.

## Ethics Statement

The study was approved by the institution review committee (Comitato Etico Interaziendale A.O.U. Città della Salute e della Scienza di Torino). Written informed consent to participate in this study was provided by the participants’ legal guardian/next of kin.

## Author Contributions

CD and DM conceived and designed the study protocol with input from FA, FR, and BV. CF, AG, LA, and BS carried out literature searches. BS, CF, VFC, and LA collected the data. DM designed and carried out the statistical analysis. CD, AG, DM, and BV interpreted the data and drafted the manuscript. FR and FA supervised the writing of the manuscript. All authors critically reviewed and contributed to the final version of the manuscript.

## Conflict of Interest

In the last 2 years, BV has received consultant fees or honoraria from Medice, Menarini, and Angelini Pharmaceuticals. FR has received sponsorship or fee for advisory board from Roche, Novartis, Biogen, PTC Therapeutics, Sanofi Genzyme, Sarepta/biogen and CD has received consultant fee from Roche and Lundbeck, and DM has received consultant fee from Ethos Ltd. The remaining authors declare that the research was conducted in the absence of any commercial or financial relationships that could be construed as a potential conflict of interest.

## Publisher’s Note

All claims expressed in this article are solely those of the authors and do not necessarily represent those of their affiliated organizations, or those of the publisher, the editors and the reviewers. Any product that may be evaluated in this article, or claim that may be made by its manufacturer, is not guaranteed or endorsed by the publisher.
